# Evidence for deficient motor planning in ADHD

**DOI:** 10.1038/s41598-017-09984-7

**Published:** 2017-08-29

**Authors:** Anat Dahan, Miriam Reiner

**Affiliations:** Virtual-Reality & NeuroCognition Lab, Technion - Israel Institute of Technology, Haifa, Israel

## Abstract

We compare motor planning mechanisms of ADHD and control subjects based on their effect on later observed kinematic characteristics. We monitor hand movement following planning conditions that differ in preparation time, and evaluate the differences across conditions and participants with/without ADHD. Our findings show that when there is sufficient planning time, people without ADHD seem to have a motor plan ready, and immediately initiate a planned movement after a ‘GO’ cue, with a bell shaped velocity profile. When planning time is not sufficient, they start the movement in a delayed time, possibly indicating that they needed to complete a movement plan. However, people with ADHD, did not start movement immediately after the cue, even when provided with a long preparation time, possibly indicating that even for this planning interval they did not have a motion plan ready. The movement was not only delayed, its velocity profile was not bell shaped and had several peaks. We further found differences between control and ADHD participants in the velocity profile, variability and jitter of movements. Our results suggest that ADHD motion characteristics, are associated with an immature motor plan. Based on the results we propose a paradigm to evaluate deficiencies in motor planning.

## Introduction

While the cognitive and behavioral Impairments in ADHD have been vastly studied, it has recently become clear that ADHD has a motor associated typicality too, and that motor abnormalities are closely linked to ADHD symptoms^1,2^. The question we raise here is whether the unique characteristics of ADHD motion patterns are rooted in deficient motor plan preparation, prior to actual execution or is it mainly correlated with processes of motor control, i.e. online monitoring of movement execution?

It has been shown that individuals who are diagnosed with ADHD have atypical motor development and behavior, as slower performance on timed motor tasks^3^, and larger speed variability^4,5^. Further difficulties were observed in locomotor skills as running and hopping, catching and throwing^6^, and deficient fine motor skills^7^. Although a variety of clinical tests have shown that a motor dysfunction is present in ADHD, they do not indicate which specific processes are involved–is it deficient motor planning or deficient motor control^8^. Dahan *et al*.^9^, suggested to examine motor deficiencies through the prism of four, partly overlapping, components: movement planning, execution, attention to task and motor control (motion monitoring). Here, we seek for a methodology that would allow identifying which of the above stages is correlated to poor motor performance – more specifically we ask whether poor execution in a motor task is a result of deficient motor control or rather it is rooted in an incomplete or immature movement planning, in the planning stage.

The underlying principles of human movement planning has been the subject of much debate, as to how much of the motor behavior is set prior to the movement execution, and how much of the behavior is determined online. An approach supporting in advance planning, has been put forward, as the motor program view^10^, suggesting that there exists an internal representation of the desired movement available before the onset of the movement. A strong support for this approach, has been demonstrated by the isochrony principle, which shows that the average velocity of point-to-point movements increases with the distance between the points^11–13^ while the total time remains the same. This implies an existence of a preexisting motor program before movement onset.

A specific subset of movements, which are subject of much interest, are object reaching movements. Such movements involve a few interleaved processes: deciding which object to reach and planning the specific parameters of the movement. A model, put forward by Cisek^14^ suggests that these are not separate processes but rather, are made within the very same neural circuits that control the execution of these actions.

Accordingly, the model suggests that sensory information in the dorsal visual stream is used to specify the spatial parameters of several currently available potential actions in parallel. These potential actions are represented by peaks of activity in corresponding fronto-parietal neural populations^15–17^. Whenever multiple peaks appear simultaneously within a single cortical region, they compete against each other through mutual inhibition, until a certain activity surpasses a threshold, and then suppresses its opponents and wins the competition. These fronto-parietal activations are is biased by a variety of influences from other regions, including the basal ganglia^18^ and the prefrontal cortex^19^ which accumulate evidence for each particular choice.

When looking at structural and functional brain differences between ADHD and normal population, there are many findings of differences in regions related to cognitive functioning, executive functioning and attention^20,21^. One of the most-studied executive control deficits in ADHD is motor inhibition^22^. Several studies of inhibitory tasks in children with ADHD show hypo-activation in fronto-parietal and fronto-striatal circuits^22–25^. Many of the differences found in the neural systems between ADHD and Normal populations are present in the areas responsible for motor control, in the premotor cortex, cerebellum and dorsal striatum^26^. There is evidence pointing towards difficulties in areas related to motor planning in ADHD. Sharma & Couture^27^, show that the prefrontal cortex, caudate and cerebellum, areas that play a role in attention, organization of thought and also motor planning, have a delay in maturation in children with ADHD^28^. Additionally, hypo functioning of the basal ganglia was found to predict difficulties in movement preparation and higher-order cognitive planning deficits^29^ all of which have been implicated in ADHD^30,31^. Hence, it is plausible to assume that there are flaws in motor planning in motor tasks performed by individuals with ADHD.

Few studies have looked at the aspect of preparation for movement in ADHD. Yan & Thomas^32^ analyzed trajectories of aiming arm movements, on the surface of a digitizer flat surface (that registered the motion pattern), of children with and without ADHD. They found that children without ADHD, had a bell shaped symmetrical velocity profile, while children with ADHD, had slower movements with multiple velocity peaks and greater variability in movements. They suggested that this may be the result of having the entire movement programmed in advance, for children without ADHD, in contrast to ADHD children who seemed to perform “on-line” monitoring and corrections. Deficient response preparation in ADHD, was reported by Klimkeit *et al*.^33^, who showed that children with ADHD were slower than typical controls with no ADHD symptoms, in reacting to a response cue by releasing a button, but did not differ in the time taken up by the actual execution of motor responses. They suggested that ADHD is characterized by slowness in motor preparation processes but not by slowness in motor execution. Eliasson *et al*.^34^, investigated the ability of children with ADHD to program and execute goal directed movements. They designed a computerized task, where Start and End positions of the movement were always visible while the curser was either visible (with-visual-feedback) or hidden (without-visual-feedback). They found that movement control was impaired for children with ADHD, especially during the without-visual-feedback condition, possibly suggesting poorer motor programming in ADHD, and higher dependency on the visual feedback. While these experiments suggest a possible underlying deficiency in motor planning, to measured movements, to our best knowledge, there is still no experiment, that manipulates the planning conditions and accordingly measures the following movements, to isolate and manipulate the parameter of movement planning.

We therefore looked for a methodology to isolate motor impairments in the motor planning component. To this end, we designed an experiment, in a highly ecological valid environment, that allows natural hand movements, and continuous high temporal and spatial monitoring of this movement, while manipulating different conditions of planning. The experiment was implemented using haptic- empowered VR-technology, that allows the feeling of holding a stylus as naturally as one holds a pen, with no or minimal restrictions on the movement, allowing a range of motion of movement pivoting at the elbow or the shoulder with an experiment setup projected to a mirror sized 82 × 55 cc, and a stylus working space of 16 × 12 cm.

In this experiment, we investigated whether individuals with ADHD differ from controls in planning of an obstacle avoidance reaching movement. If the movement is planned, participants, on cue, can immediately begin a reaching movement with a bell-shaped velocity profile^35,36^. If there is no sufficient time for planning the movement differences can be found in times of starting the movement and in the following velocity profile. We used a paradigm introduced by Hening, Favilla and Ghez at 1988^37^, and a variation proposed by Kohen *et al*.^38^. The paradigm was used to separate between two planning conditions, in order to check if individuals with ADHD have similar differences under the two planning conditions as control subjects, or if they do not exhibit these differences, indicating a deficiency in motor planning.

## Methods

### Participants

The study was approved by the Technion ethics committee, and in accordance to guidelines of the Technion “Regulations Concerning Ethical Rules of Conducting Research Studies in Behavioral Sciences on Humans”. Informed consent was received from all participants.

Experiment was conducted on a total of 25 subjects, 12 control subjects, aged 27–50, (5 female, 7 male), and 13 ADHD subjects, ages 22–38, (5 female, 8 male). All ADHD subjects had a previous professional neurological diagnosis. Diagnosis was further verified by subjects answering the adult ADHD Self-Report Scale (ASRS-v1.1) Symptom Checklist. 7 Subjects were of an inattentive subtyped, and 6 of a combined subtype. ADHD participants on medication discontinued their medication at least 24 hour prior to testing^39^.

Inclusion criteria included: Age over 18, clinical diagnosis of ADHD made by a clinician recognized by the Technion Institute of technology.

### Apparatus

In order to allow minimal restrictions on the movement, and high ecological validity, we tested motor preparation and kinematics in a 3D virtual environment, as subjects held a stylus that allows feedback and high resolution hand position measurements. The experimental environment was composed of a projector (resolution of 1280 × 720 with refresh rate of 120 Hz) a semi-transparent horizontal mirror of size (82 W × 55 H cm) and a Desktop Phantom haptic device (also called TouchTM X) manufactured by Geomagic (Geomagic, 2014). The Desktop Phantom provided a sense of touch, and a feeling as if the user is moving on a real surface, by applying force and friction. It is composed of four links and three joints, and is able to move in working space of size (16 W × 12 H × 12 D cm).

In the experiment, the participants looked downward at the semi-transparent horizontal mirror and viewed a virtual environment projected from the screen above, and reflected in the mirror. They controlled the movement by manipulating the pen-like stylus of the Phantom robot arm, which was placed under the mirror surface. The Phantom and subject’s arm were both invisible to the subject. The Phantom exerted forces on the participant’s arm to create the sensation of natural movement over a physical surface with friction.

### Procedure and task

Our experimental design is based on a timed response paradigm introduced by Hening, Favilla and Ghez at 1988^37^ and Favilla, Henning and Ghez^40^, and a variation introduced by Kohen *et al*.^38^. Henning *et al*., trained subjects to initiate impulses of isometric elbow force at the last of a series of tones, while targets were presented with unpredictable direction, and required amplitude of the motion, at random times of 0–400 ms before the last tone. They found that at short intervals, of less than 100 ms, the direction of the response was unrelated to the target. As the available time before the last tone increased, the proportion of correct directions of movement gradually increased, and correct specification was complete at intervals of over 300 ms.

**Figure 1:**
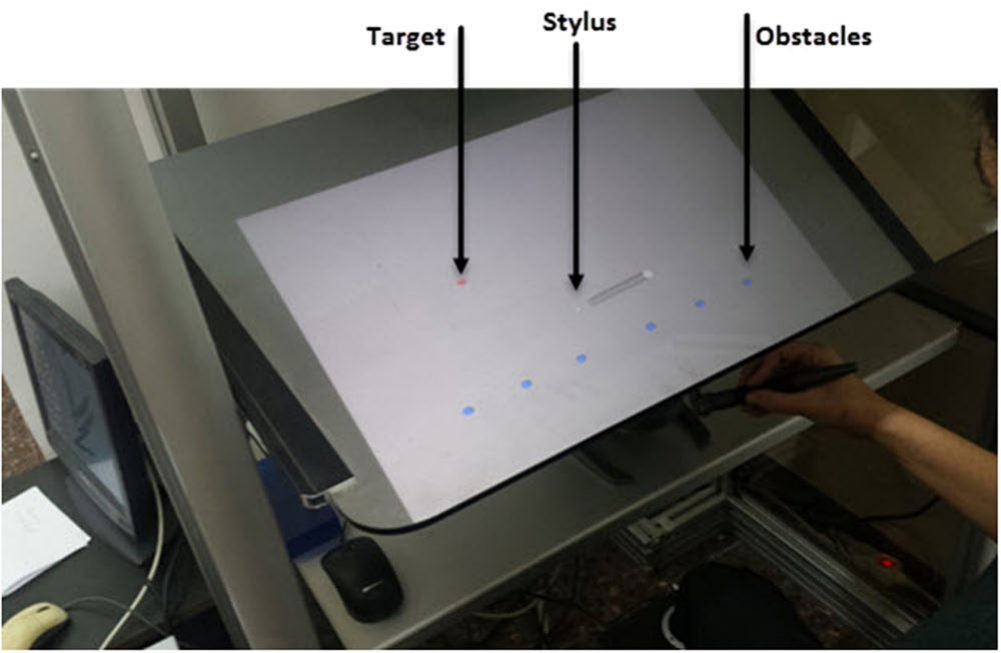
Experimental Design. A player performing the experiment. Participants initiate obstacle-avoidance (obstacle are seen as blue dots) movements leading the stylus from the starting point toward the target (in red). They control the movement, in the virtual environment, by manipulating the pen-like stylus of the Phantom robotic arm.

**Figure 2:**
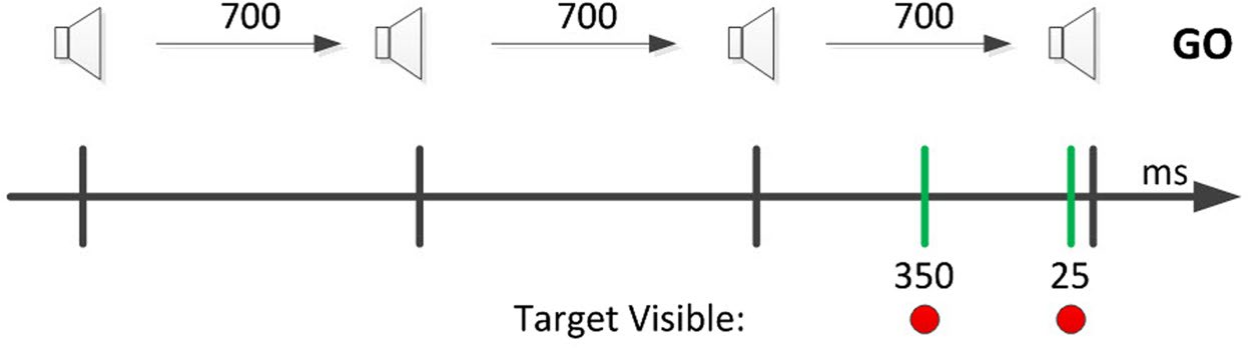
Experimental timing illustration. Subjects were trained to leave the starting point at the last in a sequence of four beeps. Beeps were separated by 700 ms intervals. Targets were presented at one of two time points: 25 ms or 350 ms prior to the final auditory ‘GO’ signal.

In the experiment, the participant held a haptic stylus device that was represented in the virtual world as a stick (Fig. 1). The initial position was set in the center of the virtual world, and served as a middle fixation point. Participants were trained to initiate a movement towards a target starting at the fourth of four consecutive tones (Fig. 2). One of four possible targets appeared at a fixed radius from the center, but at different spatial angels. At each trial, one of the four targets was selected randomly. The selected target appears at one of two time intervals before the last tone:

1. Short: d = 25 milliseconds (msec) before the last tone. 25 msec is below the time required for humans to complete movement planning.
2. Long: d = 350 msec before the last tone. This interval is long enough for control subjects to plan a movement.

Selected time intervals were based on the findings of Favilla Henning and Ghez^40^. An interval of 25 ms, well below 100 ms, fits the finding of an interval where the direction of the response was unrelated to the target. An interval of 350, fits intervals above 300 ms where correct specification was complete.

As the forth tone was heard the player led the stylus towards the target while avoiding the obstacles on the way.

Each of the four targets appears at random order, 18 times for each planning condition, resulting in 18*4(number of targets)*2(planning condition) = 144 trials.

Each player performed up to ten practice trials before the test, to get used to initiating movement at the fourth beep, and not before.

### Data analysis

Tracking data was measured for each trial at a sampling rate of 120 Hz. Data was further analyzed using MATLAB (The Mathworks, Inc., Natick, Massachusetts). Tracking data was smoothed using an sgolayfilter^41^. After soothing, data was derived to calculate velocity. Jitter was evaluated with a bandpass filter of the relevant segment of velocity (v > 0) centered at 1.5 Hz, divided by rms (root mean square) of the total velocity. This measure is an indication to the ratio of velocity, with 1–2 hz fluctuations, in relation to all velocity.

### Statistical analysis

For comparisons between groups and between experimental conditions, anova with repeated measures was adopted. A series of 2 × 2 (Control-ADHD x ShortPlanning-LongPlanning) mixed-model ANOVAs tested for effects of planning interval, and type of subject for different performance factors. For further analysis of specific conditions, we performed paired t-test to compare between short-long planning conditions, or a two sample t-test, to compare between ADHD-Control subject types. All statistical analysis was done using JMP, Statistical analysis™, from SAT, data analysis software.

### Data Availability

The datasets generated during and/or analyzed during the current study are available from the corresponding author on reasonable request.

## Results

We compared motor execution for non-ADHD (control) subjects under two conditions: short and long planning time. The planning time was determined by a short, 25 ms, time gap between the appearance of the target and the GO cue, and long, 350 ms, between the appearance of the target and the GO cue, to see which aspects of the movement are affected by the gap in planning time. We then compared motor execution for ADHD subjects under the same conditions, to identify the general difference in execution, and difference in the effect of longer planning time between the ADHD and the control group. A series of 2 × 2 (Control-ADHD x ShortPlanning-LongPlanning) mixed-model ANOVAs tested for effects of planning interval, and type of subject for five performance factors: time of start of movement, variability in time of start of movement, time of max speed, value of max speed, jitter in movement.

The effect of planning interval was significant for time of start of movement with a significance of p < 0.01, F = 108.45, significance of subject type p = 0.027, F = 5.5, Interaction p = 0.0005, F = 16. The effect of planning interval was significant for variability in time of start of movement with a significance of p = 0.0092, F = 8.08, significance of subject type p = 0.016, F = 6.75, Interaction p = 0.027, F = 5.55. The effect of planning interval was significant for time of max velocity with a significance of p = 0.0065, F = 9.05, significance of subject type p = 0.0005, F = 6.45, Interaction p = 0.019, F = 6.32. Max Speed was only significant in relation to subject type with p = 0.05, F = 4.01. Jitter was only significant in relation to subject type with p = 0.04, F = 4.54. For further analysis of specific conditions, we performed paired t-test to compare between planning conditions, or a two sample t-test, to compare between subject types.

### Profile of start time, and velocity after cue of control subjects under the short and long planning condition

**Figure 3:**
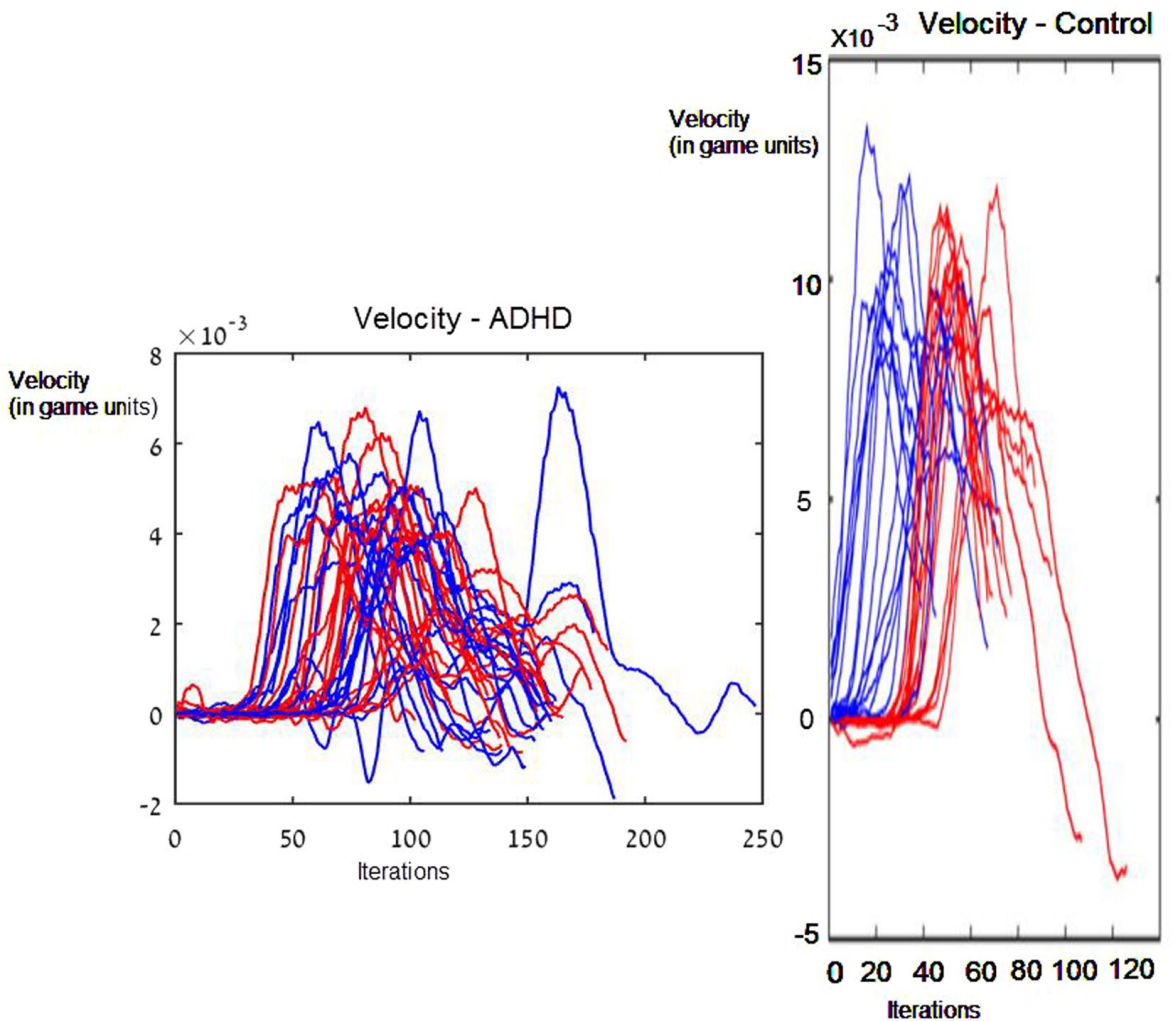
Velocity profile. ADHD vs Control. Left: Velocity profile for ADHD subject s.g. Blue: long planning condition. Red: short planning condition. Right: Velocity profile for control subject n.s Blue: long planning condition. Red: short planning condition.

**Figure 4:**
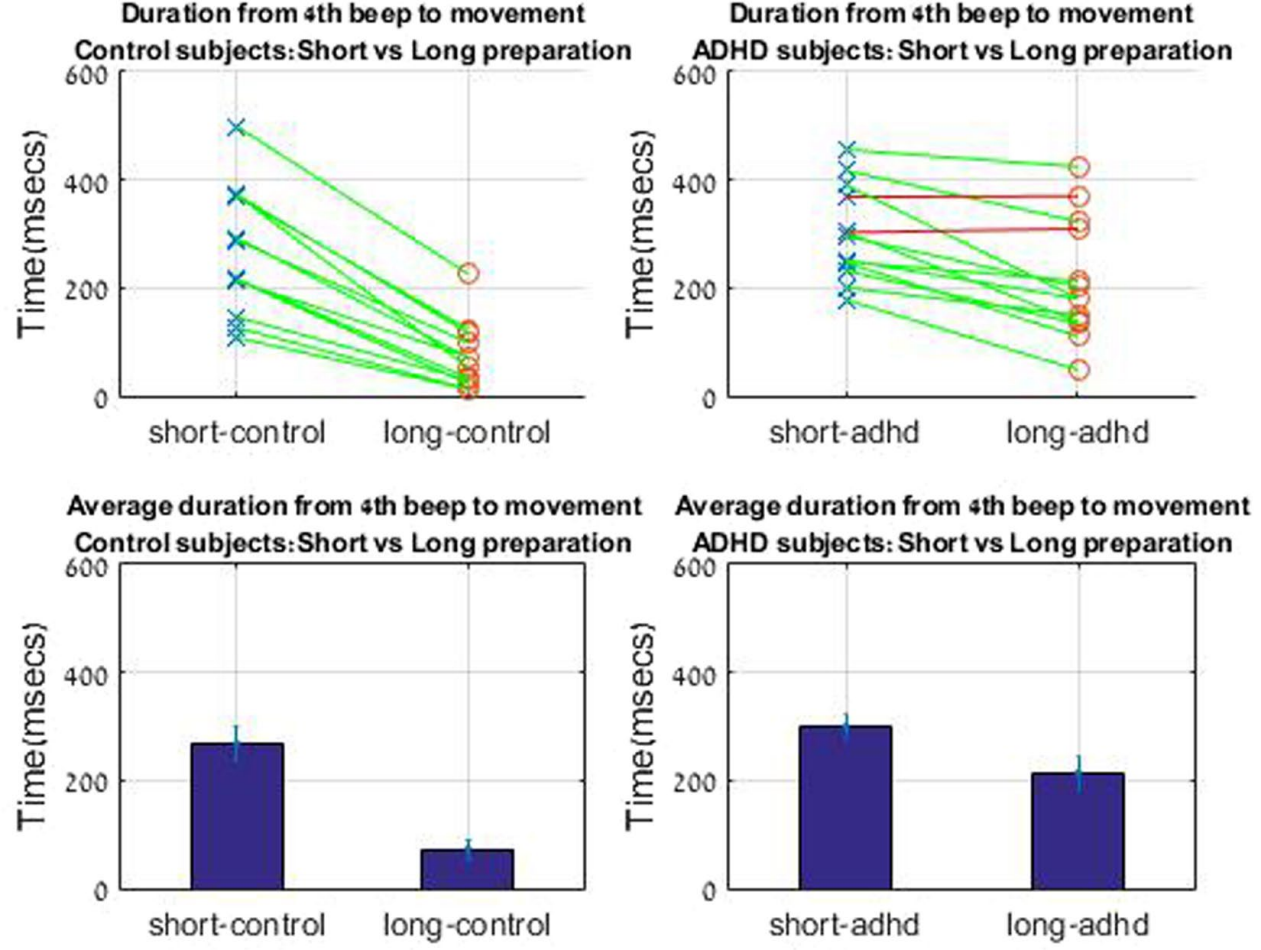
Start time. Start time of movement for different conditions for control and ADHD subjects. Control subjects started movement shortly after the cue in the long planning condition. This did not occur for ADHD subjects. Each line connects the values of start time for long and short durations for each subject.

**Figure 5:**
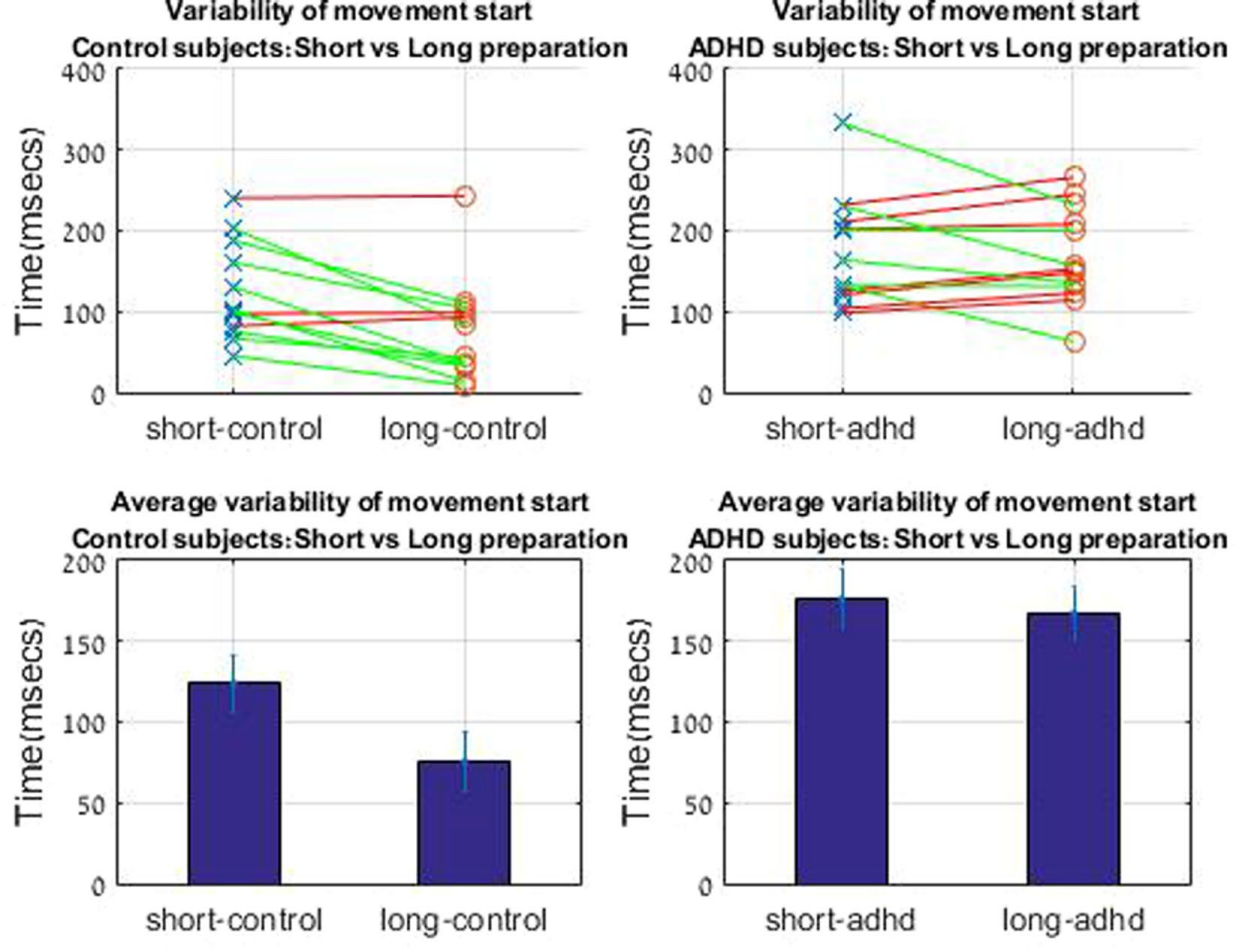
Variability of start time. Variability of start time within subject trials. Intrasubject variability was reduced in the long planning condition. This did not occur for ADHD subjects, where intrasubject variability was generally higher. Each line connects the values of variability in start time for long and short durations for each subject.

Under the short planning condition, of 25 ms between the appearance of a target and forth beep, the onset of the movement was delayed. A typical plot of velocities, plotted for all trials for one subject can be seen in Fig. 3. We defined start of movement when the velocity of the stylus passed a threshold of v > 0.0001 (in game units). In the short planning condition movement started on average 283.7 msec after the fourth beep, and in the long planning condition after 84.4 msec (Fig. 4). The effect of the planning condition was significant with p < 0.01 for the start time measure (t(12) = −4.77,p < 0.01). Difference was also found in intra-subject variability, as calculated by the standard error of the start time between the two conditions between the different trials. In the long planning condition, variability of starting time between trials was significantly reduced by 60% compared to the short planning condition (t(11) = −4.09,p < 0.01) (Fig. 5).

**Figure 6:**
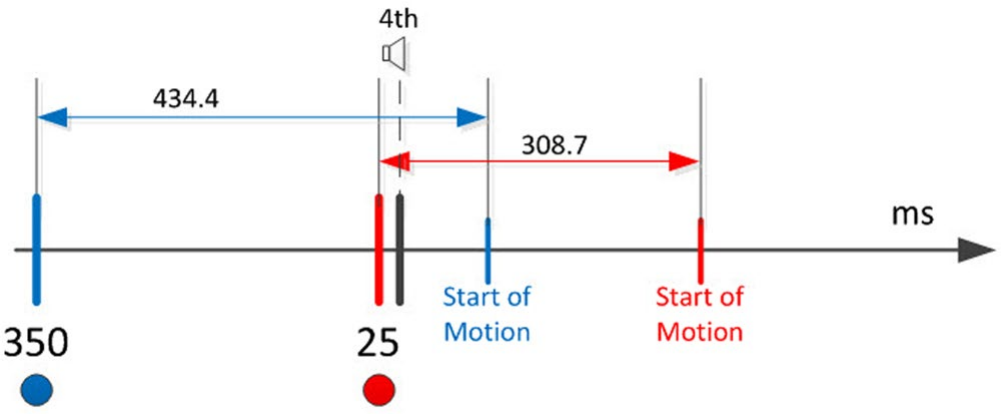
Timing Diagram. Timing from appearance of target to start of the participant’s hand motion in the short and long conditions. Blue: long planning condition. Red: short planning condition. Planning time is available before the 4th beep. Further time is obtained after the beep and before actual movement.

In the two conditions, we have a difference in available planning time before the beep, which results in a smaller difference in overall planning time. Although movement is delayed, there is still a gap in time between appearance of target and the beginning of movement: In the short preparation condition; 25 ms before the fourth beep + 283.7 ms after beep = 308.7 msec. In the Long preparation condition 350.7 ms before fourth beep + 84.4 ms after beep = 434.4 ms. (Fig. 6).

It seems that for control subjects, the difference in overall planning time, or the difference of planning before or after the GO cue, leads to differences in the velocity profile of the movement following the cue. When the profile of a velocity takes the shape of a bell shaped velocity profile, it may be concluded that the movement was programmed before the initiation of the movement^35,36^, possibly as a policy for achieving minimal jerk^11^.

The velocity profile for the long planed movements had a more narrow “bell shape”, as measured by the time from beginning of movement (Tstart) to the time of maximal velocity (Tmax), with an average of Tmax-Tstart = 362.005 msec. For control subjects, short planned movements had a wider “bell shape”, as measured by the time of the duration from the start of movement to the time of maximal velocity, with an average of Tmax-Tstart = 424.17 msec(t(11) = −4.09, p < 0.01).

This gap, between the two planning conditions, in both the time needed to start the movement after the cue, and in the duration of movement, results in differences in total execution time. The ratio between the execution time between the long and short planning condition was calculated by averaging execution time for each subject, and then averaging for the whole group, resulting in a ratio of: TTshort/TTlong = 1.41. We will now test whether the same profiles can be identified in ADHD, and specifically look at the difference in characteristics of the ADHD/noADHD groups, for each condition.

### Velocity profile of ADHD subjects

The velocity pattern in ADHD subjects reveals interesting differences from control subjects. There is a significantly smaller difference in the movement start time between the short and long planning conditions. Moreover, the start time in both conditions was delayed relative to the control’s long planning condition. In the short planning condition movement started 299.239 msec after the forth beep. In the long planning condition, movements started 213.831 msec after the forth beep (Fig. 4). Differences in start time, between control and ADHD, after the long planning conditions was significant t(19) = −3.9, p < 0.01.

For control subjects, the variability of start time within subject trials was reduced in the long planning condition. This did not occur for ADHD subjects, where intra-subject variability is similar for both conditions (p = 0.44) and generally higher than for control subjects (Fig. 5), the difference between variability the long planning conditions was significant between groups, (t(22) = −3,7, p < 0.01). Difference between groups was not significant after the short planning condition (t(22) = −1.6, p = 0.1).

However, velocity is not just delayed in a manner similar to the control subjects’ short planning condition. Rather, velocity profile of ADHD looks different and is hardly bell shaped (Fig. 3).

Thus, two variables characterize motor changes in ADHD: delay in starting motion and velocity profile. Timing of max velocity from beginning of movement is very much delayed in comparison to control subjects (t(31) = −3.6, p < 0.01). In the short planning condition, the time of max velocity is 588.771 msec after beginning of movement. In the long planning condition, 568.691 msec.

Movement for ADHD subjects were also slower than control subjects. Average Max velocity was 0.00489 in the short planning condition and 0.00516 in the long planning condition (in the experiment dimension units). While for control subjects, Average Max velocity was 0.00750 in the short condition and 0.00753 in the long condition. Difference was significant between groups in both conditions, for both the short planning condition (t(13) = 2, p = 0.03) and the long planning condition, (t(13) = 1.8, p = 0.04).

These smaller timing differences of the ADHD participants compared to controls, in beginning of movement timing and in duration of movement, between the two planning conditions result in a smaller gap in total execution time. The ratio between the execution time between the long and short planning condition was calculated by averaging execution time for each subjects, and then averaging for the whole group, resulted in a ratio of: TTshort/TTlong = 1.14 compared to 1.41 for control subjects.

### Jitter for ADHD subjects

Jitter is an indication of online corrections of the movements due to overshoots and undershoots. Jittery motion has been found in the high frequencies of 1–2 HZ in studies of manual tracking^42^, where in the absence of feedback for correction, players performed smoother tracking and greatly reduced the signal power of their responses between 0.5–1.8 Hz. Analysis of frequencies in the 2–3 hz domain was used by Noy *et al*.^43^, in a leader-follower task. They found that the follower, rather than lagging behind the leader, overshoots and undershoots the leader’s motion with a characteristic frequency of 2–3 hz. These frequencies indicate online corrections typical healthy subjects.

Similarly, in our analysis, the jitter of each player was quantified by the relative Fourier root-mean-square (rms) power in the 1–2 Hz band^43^.

Jitter in the movement of ADHD subjects was almost twice as high than that of control subjects, with values of 0.007134, and 0.007404, for the short and long conditions (t(21) = −2.3, p = 0.02, in the short condition, t(23) = −1.7, p = 0.04, in the long condition).

To summarize, for control subjects we found differences between the short and long planning conditions in start time of movement, intra-subject variability, maximum speed, and duration from start to maximum velocity. These differences, between responses to short and long planning conditions, were reduced for ADHD subjects. Moreover, movement of ADHD subjects was generally more delayed, slower, with increased intra-subject variability and larger jitter (Table 1).

**Table 1:** Summary of results for ADHD and control under the two planning conditions.

	Planning interval before beep	Delay between beep and movement	Duration from start to max velocity	Max Speed	Jitter	short to Long Delay Ratio
Control-Short Planning	25	283.7 ± 34.3	424.17 ± 52.5	0.0075 ± 0.0032	0.00382 ± 0.0007	
Control-Long Planning	350	84.4 ± 17.8	362 ± 44.1	0.00753 ± 0.0031	0.004427 ± 0.001	0.296
ADHD - Short Planning	25	299.239 ± 23.8	588.771 ± 59.1	0.00489 ± 0.0002	0.007134 ± 0.001	
ADHD-Long Planning	350	213.831 ± 30.6	568.691 ± 58.7	0.00516 ± 0.0009	0.007045 ± 0.001	0.714

## Summary and Discussion

In this study, we found substantial differences between ADHD and non ADHD subjects in motor execution such as: higher variability in velocity, difference in response time, and jitter. The kinematics of motion, and differences in patterns of motion are often absent in ADHD literature. The reason seems to be related to technical constraints—it was almost impossible to collect fine-grain, high resolution data on natural motion, in an ecological valid experimental environment. Recent technologies of immersive, touch-enabled virtual reality provide the tools needed to build a highly valid experimental setup. Thus, here we designed an experimental setup and protocol to identify the differences in motion between ADHD and non, and specifically ask whether the differences in motion execution patterns are rooted in the online monitoring of the motion or in the motor preparation stage. Our goal is to pinpoint the underlying mechanism that correlates with the deviation of ADHD motor execution: is it the preparatory neural network or the online monitoring system? Thus we examine RT and kinematics after a manipulation in available planning time, and look for the characteristics of ADHD movements in a natural, ecological valid environment.

In control subjects, there are clear differences in kinematics and response times between the short planning and long planning conditions. The motor program view, which suggests an existence of an internal representation of the desired movement, available before movement onset^10^, has much support in the literature^12,13^. Specifically, goal directed kinematics following different planning conditions, may be explained under the framework of the affordance competition hypothesis^14^. We interpret our finding in light of this framework. The experiments include a target repeatedly appearing in one of four possible locations. Accordingly populations for four, partly overlapping movement plans are active. As a target appears, it is processed by the ventral stream with interaction with the basal ganglia, and the corresponding neural population activity is enhances while the others are inhibited. Upon the auditory go cue, the population activity is above threshold and movement is initiated. In the short planning condition, when timing is insufficient, we interpret out finding in the following manner. Four, partly overlapping movement plans are active. The target appears, and is processed by the ventral stream with interaction with the basal ganglia. The go cue is played before ventral and basal ganglia processing is complete, therefore threshold is reached at a delay after go cue, and movement is initiated with a delay.

However, in ADHD subjects a smaller difference between the two planning interval conditions can be found and movement starts with a delay compared to controls in both conditions. We try to understand this behavior in light of the affordance competition hypothesis. The delay in start times in relation to healthy subjects may indicate a delay in reaching the threshold, due to deficient modulation of attentional processes in the ventral stream. Moreover, the following ADHD movements were more variable, with multiple velocity peaks and more jitter, possible indicating less inhibition of competing movement plans, modulated by the basal ganglia, resulting in a less planned movement, and interference of competing movement plans.

This model implies that the deficiencies found in ADHD planning are not related to a flaw in a specific region or functionality, but rather are related to modulation and connection between different regions of the brain.

The subject of functional and structural connectivity in ADHD has lately been a subject of much interest, asking if the ADHD brain is wired differently. Some studies raise questions of the role of functional/structural connectivity during resting and task states, and point at convergent evidence for white matter pathology and disrupted anatomical connectivity in ADHD^44^. The causal link between disturbed white matter connectivity and cortical/behavioral dysfunction is not clear and might originate in multiple factors (Ibid). Looking at the difference in the activations of the default-mode network (DMN), in ADHD and non, might suggest additional directions. The ‘default-mode’ attention network is a set of brain regions, primarily located along the medial wall of the brain, including the posterior cingulate, precuneus, and middle temporal gyrus. These regions are associated with task-irrelevant mental processes that are putatively *suppressed* in order to perform optimally in cognitively demanding situations^45^. This system, and specifically the precuneus/PCC are involved in visuospatial processing, and direction attention in space such as when moving an object in space to hit a target, or when preparing for the movement^46^, and motor coordination^47^. The MPFC is implicated in motor memory consolidation^48^ and the medial, lateral, and inferior parietal cortex is involved also in real time sensory processing such as during motion, for controlling the movement.

While the DMN is task irrelevant, a second system that includes the dorsolateral prefrontal cortex (DLPFC), the intraparietal sulcus (IPS), and the supplementary motor area (SMA), is task relevant, i.e. activated in task states more than in task irrelevant states, and associated with increased alertness motor response and control^46^. The task relevant and task irrelevant systems are linked in opposite attenuation –activation. When one is highly active the other is attenuated. A recent approach assumes that ADHD may be related to disturbances in communications across brain areas that are part of the DMN. Indeed disrupted or modified communications across areas might affect the DMN, resulting in reduced capability to modulate itself, and hence interferes with attentional and motor tasks, and specifically in the process of having a motor plan surpass threshold and ready to GO.

### Conclusions

Different planning intervals lead to differences in timing of initiation of movement, and of the corresponding velocity profile. These differences are diminished for individuals with ADHD. We suggest that these diminished differences indicate poor motor planning in individuals with ADHD.

We suggest to consider this methodology as a test for deficiencies in motor planning. Such a test needn’t be specific for ADHD and may be useful for other cognitive conditions that may result in motor planning impairments, such as following brain injury, and on the autism spectrum.

We suggest to further research this methodology, by using brain imaging techniques to correlate between the differences in the planning conditions between control and ADHD subjects, to differences in brain activations.

## References

[1] Kaiser ML, Schoemaker MM, Albaret JM, Geuze RH (2015). What is the evidence of impaired motor skills and motor control among children with attention deficit hyperactivity disorder (ADHD)? *Systematic review of the literature*. Research in developmental disabilities.

[2] Gilbert DL, Isaacs KM, Augusta M, Macneil LK, Mostofsky SH (2011). Motor cortex inhibition A marker of ADHD behavior and motor development in children. Neurology.

[3] Piek JP, Pitcher TM, Hay DA (1999). Motor coordination and kinaesthesis in boys with attention deficit–hyperactivity disorder. Developmental medicine & child neurology.

[4] Castellanos FX, Tannock R (2002). Neuroscience of attention-deficit/hyperactivity disorder: the search for endophenotypes. Nature Reviews Neuroscience.

[5] Gilden DL, Hancock H (2007). Response variability in attention-deficit disorders. Psychological Science.

[6] Harvey WJ (2009). Physical activity experiences of boys with and without ADHD. Adapted Physical Activity Quarterly.

[7] Pitcher TM, Piek JP, Hay DA (2003). Fine and gross motor ability in males with ADHD. Developmental medicine and child neurology.

[8] Sergeant JA, Piek JP, Oosterlaan J (2006). ADHD and DCD: A relationship in need of research. Human Movement Science.

[9] Dahan, A., Ryder, C. H., & Reiner, M. Components of motor deficiencies in ADHD and possible interventions. *Neuroscience*. (2016). 10.1016/j.neuroscience.2016.05.040 27235737

[10] Viviani P, Flash T (1995). Minimum-jerk, two-thirds power law, and isochrony: converging approaches to movement planning. Journal of Experimental Psychology: Human Perception and Performance.

[11] Fitts PM (1954). The information capacity of the human motor system in controlling the amplitude of movement. Journal of experimental psychology.

[12] Viviani P, McCollum G (1983). The relation between linear extent and velocity in drawing movements. Neuroscience.

[13] Viviani P, Schneider R (1991). A developmental study of the relationship between geometry and kinematics in drawing movements. Journal of Experimental Psychology: Human Perception and Performance.

[14] Cisek P (2007). Cortical mechanisms of action selection: the affordance competition hypothesis. Philosophical Transactions of the Royal Society of London B: Biological Sciences.

[15] Platt ML, Glimcher PW (1997). Responses of intraparietal neurons to saccadic targets and visual distractors. Journal of neurophysiology.

[16] Cisek P, Kalaska JF (2004). Neural correlates of mental rehearsal in dorsal premotor cortex. Nature.

[17] Cisek P, Kalaska JF (2005). Neural correlates of reaching decisions in dorsal premotor cortex: specification of multiple direction choices and final selection of action. Neuron.

[18] Redgrave P, Prescott TJ, Gurney K (1999). The basal ganglia: a vertebrate solution to the selection problem?. Neuroscience.

[19] Tanji J, Hoshi E (2001). Behavioral planning in the prefrontal cortex. Current opinion in neurobiology.

[20] Biederman J (2005). Attention-deficit/hyperactivity disorder: a selective overview. Biological psychiatry.

[21] Makris N, Biederman J, Monuteaux MC, Seidman LJ (2009). Towards conceptualizing a neural systems-based anatomy of attention-deficit/hyperactivity disorder. Developmental neuroscience.

[22] Castellanos FX, Proal E (2012). Large-scale brain systems in ADHD: beyond the prefrontal–striatal model. Trends in cognitive sciences.

[23] Dickstein SG, Bannon K, Xavier Castellanos F, Milham MP (2006). The neural correlates of attention deficit hyperactivity disorder: An ALE meta‐analysis. Journal of Child Psychology and Psychiatry.

[24] Castellanos FX, Sonuga-Barke EJ, Milham MP, Tannock R (2006). Characterizing cognition in ADHD: beyond executive dysfunction. Trends in cognitive sciences.

[25] Rubia K (2011). “Cool” inferior frontostriatal dysfunction in attention-deficit/hyperactivity disorder versus “hot” ventromedial orbitofrontal-limbic dysfunction in conduct disorder: a review. Biological psychiatry.

[26] Demers, M. M., McNevin, N. & Azar, N. R. ADHD and motor control: a review of the motor control deficiencies associated with attention deficit/hyperactivity disorder and current treatment options. *Critical Reviews™ in Physical and Rehabilitation Medicine*, **25****(****3–4****)** (2013).

[27] Sharma A, Couture J (2014). A review of the pathophysiology, etiology, and treatment of attention-deficit hyperactivity disorder (ADHD). Annals of Pharmacotherapy.

[28] Cortese, S. *et al*. Toward systems neuroscience of ADHD: a meta-analysis of 55 fMRI studies. *American Journal of Psychiatry* (2012). 10.1176/appi.ajp.2012.11101521 PMC3879048 22983386

[29] Bradshaw, J. L., & Mattingley, J. B. Clinical neuropsychology: Behavioral and brain science. (Elsevier, 2013).

[30] Barkley RA (1991). The ecological validity of laboratory and analogue assessment methods of ADHD symptoms. Journal of abnormal child psychology.

[31] Van der Meere, J. J. The role of attention in *Hyperactivity and attention disorders of childhood* (ed. S. Sandberg) 162–213 (Cambridge University Press, 2002).

[32] Yan JH, Thomas JR (2002). Arm movement control: differences between children with and without attention deficit hyperactivity disorder. Research quarterly for exercise and sport.

[33] Klimkeit EI, Mattingley JB, Sheppard DM, Lee P, Bradshaw JL (2005). Motor preparation, motor execution, attention, and executive functions in attention deficit/hyperactivity disorder (ADHD). Child Neuropsychology.

[34] Eliasson A-C, Rösblad B, Forssberg H (2004). Disturbances in programming goal-directed arm movements in children with ADHD. Developmental Medicine and Child Neurology.

[35] Brooks V.B. How are “Move” and “Hold” Programs Matched?. In Cerebellar Functions. (ed. Bloedel J.R., Dichgans J., Precht W.) 1–23 (Springer, Berlin, Heidelberg, 1984).

[36] Ghez C, Hening W, Favilla M (1990). Parallel interacting channels in the initiation and specification of motor response features. Attention and performance.

[37] Hening W, Favilla M, Ghez C (1988). Trajectory control in targeted force impulses. Experimental Brain Research.

[38] Kohen D, Karklinsky M, Meirovitch Y, Flash T, Shmuelof LS (2017). The effects of shortening preparation time on the execution of intentionally curved trajectories: optimization and geometrical analysis. Frontiers in Human Neuroscience.

[39] Leitner Y, Doniger GM, Barak R, Simon ES, Hausdorff JM (2007). A novel multidomain computerized cognitive assessment for attention-deficit hyperactivity disorder: evidence for widespread and circumscribed cognitive deficits. Journal of child neurology.

[40] Favilla M, Hening W, Ghez C (1989). Trajectory control in targeted force impulses. Experimental Brain Research.

[41] Savitzky A, Golay MJ (1964). Smoothing and differentiation of data by simplified least squares procedures. Analytical chemistry.

[42] Miall RC, Weir DJ, Stein JF (1993). Intermittency in human manual tracking tasks. Journal of motor behavior.

[43] Noy L, Dekel E, Alon U (2011). The mirror game as a paradigm for studying the dynamics of two people improvising motion together. Proceedings of the National Academy of Sciences.

[44] Konrad K, Eickhoff SB (2010). Is the ADHD brain wired differently? A review on structural and functional connectivity in attention deficit hyperactivity disorder. Human brain mapping.

[45] Raichle ME (2001). A default mode of brain function. Proceedings of the National Academy of Sciences.

[46] Cavanna AE, Trimble MR (2006). The precuneus: a review of its functional anatomy and behavioural correlates. Brain.

[47] Wenderoth N, Debaere F, Sunaert S, Swinnen SP (2005). The role of anterior cingulate cortex and precuneus in the coordination of motor behaviour. European Journal of Neuroscience.

[48] Reiner M, Rozengurt R, Barnea A (2014). Better than sleep: theta neurofeedback training accelerates memory consolidation. Biological psychology.

