# Author Correction: Evidence for deficient motor planning in ADHD

**DOI:** 10.1038/s41598-018-23237-1

**Published:** 2018-04-24

**Authors:** Anat Dahan, Miriam Reiner

**Affiliations:** Virtual-Reality & NeuroCognition Lab, Technion - Israel Institute of Technology, Haifa, Israel

Correction to: *Scientific Reports* 10.1038/s41598-017-09984-7, published online 29 August 2017

The original version of this Article contained errors in the spelling of the authors Anat Dahan and Miriam Reiner which were incorrectly given as Dahan Anat and Reiner Miriam respectively.

These errors have now been corrected in the PDF and HTML versions of the Article.

